# Uncovering a Glaucoma-Linked Lysophosphatidic Acid–MAPK/AP-1 Fibrosis Axis in Human Trabecular Meshwork Cells and Its Modulation by *Diospyros kaki* Leaf Extract

**DOI:** 10.3390/ijms27031544

**Published:** 2026-02-04

**Authors:** Youngsic Jeon, Hyukjoon Kwon, Hong Ryul Ahn, Gyuwon Huh, Taejung Kim, Young-Tae Park, Hyun Bong Park, Jin-hyoung Jeong, Jae-hyun Jo, Young-Joo Kim, Sang Hoon Jung

**Affiliations:** 1 Center for Natural Product Efficacy Optimization, Korea Institute of Science and Technology (KIST), Gangneung Institute of Natural Products, Gangneung 25451, Republic of Korea; biomangg0@kist.re.kr (Y.J.);; 2 R&D Center, NovMetaPharma Co., Ltd., Seoul 06050, Republic of Korea; 3 Natural Product Applied Science, KIST School, University of Science and Technology, Gangneung 25451, Republic of Korea; 4 Department of Biology, College of Natural Sciences, Gangneung-Wonju National University, Gangneung 25457, Republic of Korea; 5 Department of Healthcare Management, Catholic Kwandong University, Gangneung 25601, Republic of Korea; 6 Department of Digital Healthcare, Catholic Kwandong University, Gangneung 25601, Republic of Korea

**Keywords:** glaucoma, trabecular meshwork, extracellular matrix, MAPK signaling, *Diospyros kaki*

## Abstract

Dysregulated extracellular matrix (ECM) deposition and epithelial–mesenchymal transition (EMT) in the trabecular meshwork (TM) contribute to glaucoma-associated fibrotic remodeling, and lysophosphatidic acid (LPA) potently induces these profibrotic responses in human trabecular meshwork (HTM) cells. We investigated whether an ethanolic extract of *Diospyros kaki* leaves (EEDK) attenuates LPA-induced fibrosis and explored the underlying mechanisms. HTM cells were stimulated with LPA and treated with ethanol-based EEDK extracts. Expression of ECM/fibrosis-related genes (*FN1*, *ACTA2*, *COL1A1*, *COL3A1*, *COL4A1*, *COL6A2*, *CCN2*) and EMT markers (*CDH2*, *VIM*, *SNAI1*) was assessed, along with cell migration using a wound-healing assay. Upstream regulatory pathways were examined via transcription factor prediction, AP-1 reporter assays, and analyses of MAPK/AP-1 signaling. Among the extracts tested, the 70% ethanol EEDK extract showed the strongest antifibrotic activity, significantly reducing LPA-induced ECM gene/protein expression and inhibiting HTM cell migration in a dose-dependent manner, whereas the 90% ethanol extract showed minimal effects. LPA robustly activated MAPK-dependent AP-1 signaling, and either pharmacologic inhibition of MAPK pathways or treatment with 70% ethanol EEDK comparably suppressed AP-1 activity and decreased downstream ECM/EMT gene expression. Thus, 70% ethanol EEDK mitigates LPA-induced TM fibrosis by inhibiting MAPK/AP-1-mediated transcription, supporting its potential as an antifibrotic strategy for glaucoma.

## 1. Introduction

Glaucoma is a chronic optic neuropathy characterized by progressive loss of retinal ganglion cells and corresponding visual field defects, with elevated intraocular pressure (IOP) being the principal modifiable risk factor for its development and progression [1]. Globally, glaucoma affected ~76 million people in 2020 and is projected to reach ~112 million by 2040. It is also a major cause of vision loss, accounting for millions of cases of blindness and moderate-to-severe visual impairment worldwide [2,3]. In primary open-angle glaucoma, IOP elevation is mainly caused by increased resistance to aqueous humor outflow (AHO) through the conventional trabecular meshwork (TM)–Schlemm’s canal pathway [4,5]. Structural and functional alterations within the TM, such as excessive accumulation of extracellular matrix (ECM), reorganization of the cytoskeleton, and changes in cellular motility, are believed to underlie this increased outflow resistance and the resultant rise in IOP [6,7]. Consequently, strategies that prevent or reverse TM fibrosis are considered promising approaches to normalize AHO and limit glaucomatous damage.

Cells in the juxtacanalicular region of the TM exhibit fibroblast-like characteristics and express a variety of contractile and ECM-associated proteins, including α-smooth muscle actin (α-SMA), several collagen isoforms, fibronectin, and laminin [8]. In glaucomatous TM, the buildup of ECM constituents such as α-SMA and fibronectin is closely associated with narrowing of the outflow channels and reduced AHO [7,9]. In addition, epithelial–mesenchymal transition (EMT)-like alterations, marked by increased expression of mesenchymal markers such as N-cadherin, vimentin, and Snail, have been linked to fibrotic remodeling and enhanced migratory behavior of TM cells [10,11]. Nevertheless, the key upstream mediators and signaling pathways that coordinate ECM deposition and EMT-like responses in TM tissue remain inadequately defined.

Lysophosphatidic acid (LPA) is a bioactive lysophospholipid present in serum, interstitial fluids, and aqueous humor, where it acts through specific G protein–coupled LPA receptors [12,13]. Growing evidence indicates that LPA contributes to the pathogenesis of glaucoma and ocular hypertension [14]. Engagement of LPA receptors activates multiple Gα subunits and downstream pathways, including mitogen-activated protein kinase (MAPK) cascades such as ERK1/2, p38, and JNK [15]. Through these signaling networks, LPA can promote cell proliferation, survival, contraction, and migration, and has been implicated as a profibrotic mediator in various organ systems. Within the anterior segment, LPA has been shown to stimulate ECM synthesis and induce cytoskeletal rearrangements in TM cells, suggesting that dysregulated LPA signaling may drive TM fibrosis and increased outflow resistance [16,17].

Natural products and herbal preparations have attracted increasing interest as complementary or adjunctive options for long-term ocular diseases, including glaucoma. Ethanolic extracts of *Diospyros kaki* (persimmon) leaves have been reported to exhibit diverse biological activities, including antioxidant, anti-inflammatory, and neuroprotective effects. Several studies have suggested that these activities are largely attributed to flavonoids and phenolic compounds abundant in the leaves, supporting their potential as bioactive plant-derived materials [18,19]. Consistent with these observations, an ethanolic extract of *Diospyros kaki* leaves (EEDK) has previously been demonstrated to protect retinal ganglion cells from oxidative stress– and excitotoxicity-induced cell death and to reduce elevated IOP in experimental glaucoma models, at least in part via modulation of soluble guanylate cyclase α1 signaling [20]. However, whether EEDK can directly influence TM fibrotic remodeling and AHO resistance, particularly under conditions of LPA-driven profibrotic signaling, has not yet been clarified.

In this study, an LPA-stimulated human TM (HTM) cell model was employed to reproduce key features of TM fibrosis, including ECM accumulation and EMT-like changes. The effects of ethanol-based EEDK preparations, with a focus on the 70% ethanol fraction, were examined with respect to LPA-induced ECM production, EMT-related marker expression, and cell migration in HTM cells. In addition, the underlying molecular pathways were investigated, with particular emphasis on MAPK/AP-1 signaling and transcriptional regulation of ECM- and fibrosis-associated genes such as *FN1* and *ACTA2*. Collectively, these analyses were conducted to assess the potential of EEDK as a novel therapeutic candidate targeting TM remodeling in glaucoma.

## 2. Results

### 2.1. Chemical Characterization of Diospyros kaki Leaf Ethanol Extract

**Figure 1:**
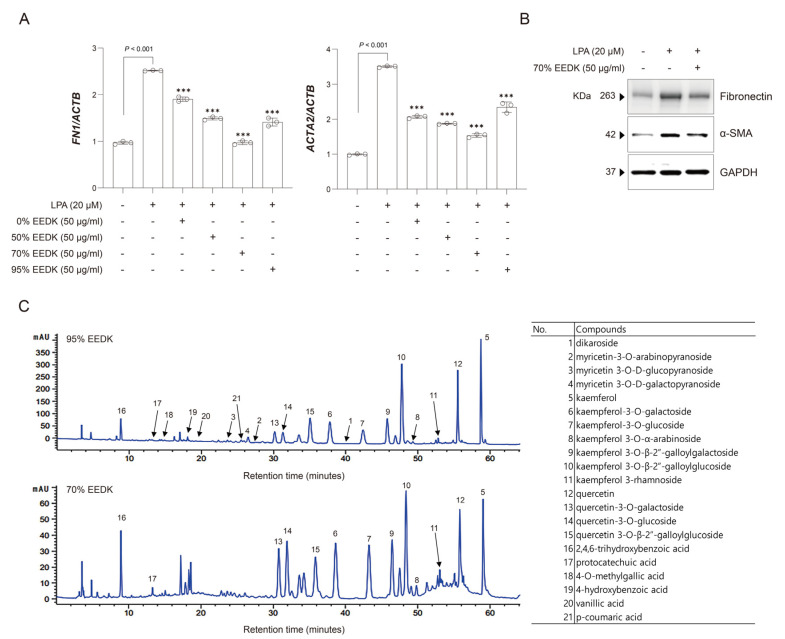
70% EEDK attenuates LPA-induced *FN1* and *ACTA2* expression in HTM cells. (**A**) Bar plots show *FN1* and *ACTA2* expression levels in HTM cells treated with vehicle control, LPA alone, and LPA in combination with EEDK extracts for 24 h. Expression of each gene was normalized to *ACTB*. Statistical significance is indicated (*** *p*  <  0.001; one-way ANOVA). (**B**) Fibronectin and α-SMA levels in HTM cells treated with LPA alone or with LPA plus the 70% EEDK extract were evaluated by Western blotting after 48 h. GAPDH was used as a normalization marker. (**C**) 95% ethanol extract of EEDK (*top*) and 70% EEDK (*bottom*) peaks were assigned as follows: (1) dikaroside, (2) myricetin-3-O-arabinopyranoside, (3) myricetin-3-O-glucopyranoside, (4) myricetin-3-O-galactopyranoside, (5) kaempferol, (6) kaempferol-3-O-galactoside, (7) kaempferol-3-O-glucoside, (8) kaempferol-3-O-arabinoside, (9) kaempferol-3-O-galloylgalactoside, (10) kaempferol-3-O-galloylglucoside, (11) kaempferol-3-rhamnoside, (12) quercetin, (13) quercetin-3-O-galactoside, (14) quercetin-3-O-glucoside, (15) quercetin-3-O-galloylglucoside, (16) 2,4,6-trihydroxybenzoic acid, (17) protocatechuic acid, (18) 4-O-methylgallic acid, (19) 4-hydroxybenzoic acid, (20) vanillic acid, and (21) p-coumaric acid.

To characterize the chemical composition of the ethanol extracts of *Diospyros kaki* leaves, high-performance liquid chromatography (HPLC) analysis was performed to compare the profiles of the 70% and 95% ethanol extracts (Figure 1C). Both extracts exhibited similar qualitative chromatographic patterns; however, clear quantitative differences were observed in the major marker compounds. The 70% ethanol extract showed higher peak intensities for several flavonoid glycosides and phenolic acid constituents compared to the 95% ethanol extract. In particular, the relative contents of kaempferol derivatives—including kaempferol (5), kaempferol-3-O-galactoside (6), kaempferol-3-O-glucoside (7), kaempferol-3-O-arabinoside (8), kaempferol-3-O-galloylgalactoside (9), and kaempferol-3-O-galloylglucoside (10)—were markedly increased in the 70% ethanol extract. In addition, quercetin and its glycosides—quercetin (12), quercetin-3-O-galactoside (13), quercetin-3-O-glucoside (14), and quercetin-3-O-galloylglucoside (15)—as well as phenolic acids such as 2,4,6-trihydroxybenzoic acid (16), protocatechuic acid (17), 4-O-methylgallic acid (18), 4-hydroxybenzoic acid (19), vanillic acid (20), and p-coumaric acid (21), were present at relatively higher levels in the 70% ethanol extract.

### 2.2. 70%. EEDK Attenuates LPA-Induced FN1 and ACTA2 Expression in HTM Cells

To determine the appropriate concentration for subsequent experiments, the cytotoxicity of EEDK ethanol extracts (0%, 50%, 70%, and 95%) was first evaluated in HTM cells. Treatment with the 0%, 50%, and 70% ethanol EEDK extracts did not significantly affect cell viability at either 24 or 48 h. In contrast, the 95% ethanol EEDK extract caused a marked reduction in cell viability at 48 h, decreasing viability by at least 40% relative to the control (Figure S1). Based on these findings, a 24 h treatment duration was selected for all EEDK extracts in subsequent experiments.

Because *ACTA2* (α-SMA) and *FN1* (fibronectin) are widely used markers of extracellular matrix (ECM) accumulation and fibrotic remodeling in the trabecular meshwork, their induction by the profibrotic stimuli lysophosphatidic acid (LPA), transforming growth factor-β (TGF-β), and dexamethasone was assessed (Figure S2). Indeed, all three stimuli upregulated α-SMA and fibronectin in human trabecular meshwork cells and were associated with ECM remodeling [21].

Based on these results, the effects of EEDK ethanol extracts on *ACTA2* and *FN1* expression were further evaluated in LPA-induced HTM cells. Among the extracts tested, the 70% ethanol EEDK extract resulted in significantly lower *ACTA2* and *FN1* expression levels than the other extracts (*p* < 0.001, Figure 1A). Furthermore, treatment with 70% ethanol EEDK extract markedly inhibited LPA-induced Fibronectin and α-SMA protein expression (Figure 1B).

Given that the 70% ethanol EEDK extract showed the most pronounced suppression of LPA-induced *ACTA2* and *FN1* expression, it was next determined whether compositional differences between extracts could account for the observed biological effects. To this end, high-performance liquid chromatography (HPLC) analysis was performed to compare the chemical profiles of the 95% and 70% EEDK extracts (Figure 1C). Both extracts exhibited similar qualitative chromatographic patterns; however, clear quantitative differences were observed in the major marker compounds. Notably, the 70% ethanol extract displayed higher peak intensities for several flavonoid glycosides and phenolic acid constituents compared to the 95% ethanol extract. In particular, the relative contents of kaempferol derivatives—including kaempferol (5), kaempferol-3-O-galactoside (6), kaempferol-3-O-glucoside (7), kaempferol-3-O-arabinoside (8), kaempferol-3-O-galloylgalactoside (9), and kaempferol-3-O-galloylglucoside (10)—were markedly increased in the 70% ethanol extract. In addition, quercetin and its glycosides—quercetin (12), quercetin-3-O-galactoside (13), quercetin-3-O-glucoside (14), and quercetin-3-O-galloylglucoside (15)—as well as phenolic acids such as 2,4,6-trihydroxybenzoic acid (16), protocatechuic acid (17), 4-O-methylgallic acid (18), 4-hydroxybenzoic acid (19), vanillic acid (20), and p-coumaric acid (21), were present at relatively higher levels in the 70% ethanol extract. Collectively, these quantitative enrichments in polyphenolic constituents in the 70% EEDK could be associated with its superior anti-fibrotic effects observed in LPA-stimulated HTM cells.

### 2.3. 70%. EEDK Suppresses Profibrotic ECM Production in HTM Cells

**Figure 2:**
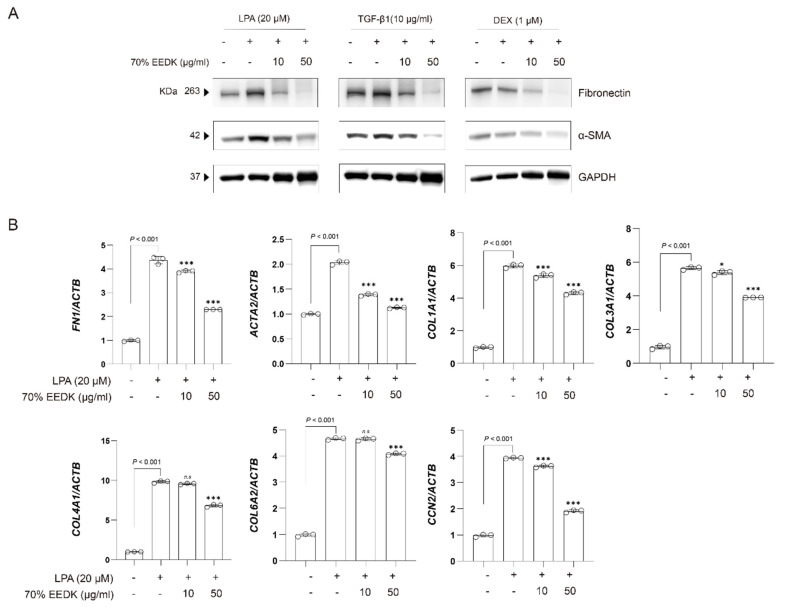
70% EEDK suppresses profibrotic ECM production in HTM cells. (**A**) Fibronectin and α-SMA levels in HTM cells treated with profibrotic stimuli (LPA, TGF-β, and DEX) in the absence or presence of 70% EEDK extract were evaluated by Western blot analysis. GAPDH was used as a normalization marker. (**B**) Bar plots show *FN1*, *ACTA2*, *COL1A1*, *COL3A1*, *COL4A1*, *COL6A2*, and *CCN2* expression levels in HTM cells treated with vehicle control, LPA alone, and LPA in combination with 70% EEDK extracts for 24 h. Expression of each gene was normalized to *ACTB*. Statistical significance is indicated (* *p*  <  0.05 and *** *p*  <  0.001; one-way ANOVA). Abbreviation: *n.s*: not significant.

To determine whether the 70% ethanol EEDK extract also suppresses ECM- and fibrosis-related proteins induced by other profibrotic stimuli, it effects on TGF-β- and dexamethasone-treated HTM cells were next examined. Similarly to its effects on LPA-induced responses, the 70% ethanol EEDK extract significantly attenuated fibronectin and α-SMA expression induced by TGF-β and dexamethasone (Figure 2A). However, these inhibitory effects were not observed with the 90% ethanol EEDK extract (Figure S3). In addition to *ACTA2* and *FN1*, the expression of other ECM- and fibrosis-related genes, including *COL1A1*, *COL3A1*, *COL4A1*, *COL6A2*, and *CCN2*, was also assessed, as these genes are involved in trabecular meshwork ECM remodeling [22]. Treatment with 70% ethanol EEDK extract reduced the expression of these genes in a dose-dependent manner (Figure 2B).

### 2.4. 70%. EEDK Inhibits LPA-Induced Cell Migration and EMT-Related Marker Expression in HTM Cells

**Figure 3:**
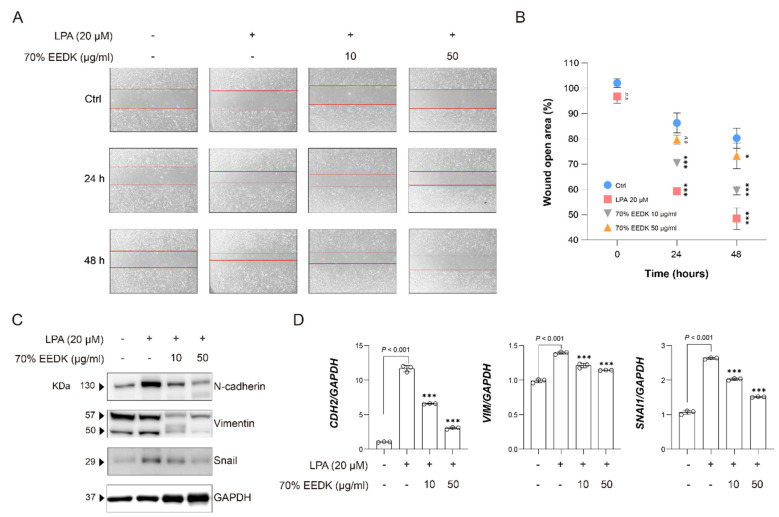
70% EEDK suppresses profibrotic ECM production in HTM cells. (**A**) Wound healing (scratch) assay in HTM cells pretreated with vehicle or 70% EEDK extracts (10 and 50 μg/mL, 2 h) and subsequently stimulated with LPA. Representative phase-contrast images were taken immediately after scratching (0 h) and at 24 h and 48 h to assess wound closure. (**B**) A point plot shows the quantification of wound closure in each treatment group, expressed as the percentage of the initial scratch area at 0 h, 24 h, and 48 h. Statistical significance is indicated (* *p*  <  0.05 and *** *p*  <  0.001; one-way ANOVA). (**C**) N-cadherin, vimentin, and snail levels in HTM cells treated with LPA and LPA with 70% EEDK extract were evaluated by Western blot analysis. GAPDH was used as a normalization marker. (**D**) Bar plots show *CDH2*, *VIM*, and *SNAI1* expression levels in HTM cells treated with vehicle control, LPA alone, and LPA in combination with 70% EEDK extracts for 24 h. Expression of each gene was normalized to *GAPDH*. Statistical significance is indicated (*** *p*  <  0.001; one-way ANOVA). Abbreviation: Ctrl: control, *n.s*: not significant.

ECM remodeling is closely linked to cell migration; therefore, the effects of the 70% ethanol EEDK extract on LPA-induced cell migration in HTM cells were next examined. In wound healing assays, LPA markedly promoted cell migration and wound closure, whereas co-treatment with the 70% ethanol EEDK extract significantly suppressed LPA-induced cell movement in a dose-dependent manner (Figure 3A,B). To investigate the underlying mechanism, the expression of migration- and EMT-related markers, including N-cadherin, vimentin, and Snail, was assessed. LPA stimulation increased N-cadherin (*CDH2*), vimentin (*VIM*), and Snail (*SNAI1*) at both the protein and mRNA levels, whereas treatment with the 70% ethanol EEDK extract attenuated these LPA-induced changes (Figure 3C,D).

### 2.5. 70%. EEDK Inhibits LPA-Induced Activation of MAPK Signaling Pathway in HTM Cells

**Figure 4:**
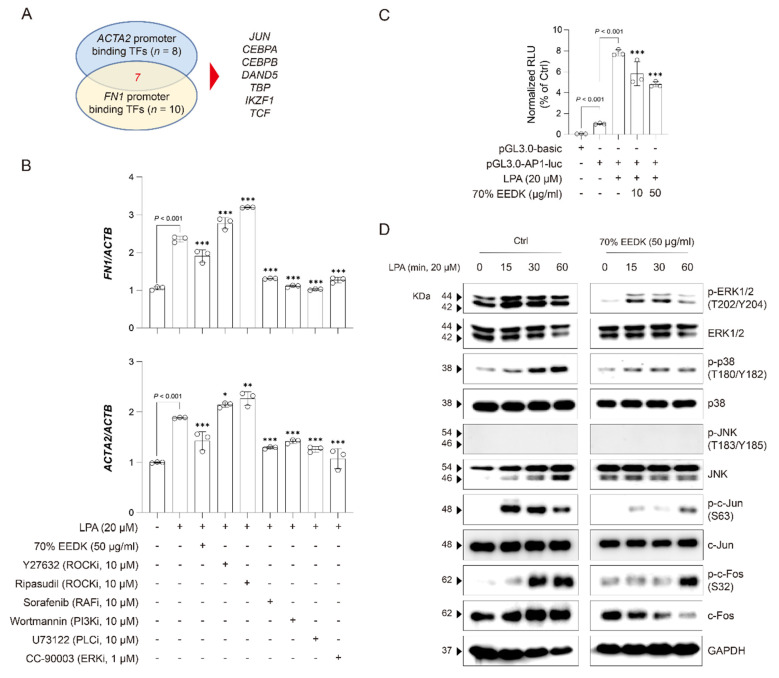
70% EEDK suppresses profibrotic ECM production in HTM cells. (**A**) Venn diagram showing the overlap of transcription factors predicted to bind the *FN1* and *ACTA2* promoters (TSS −1000 bp to +100 bp; for details, see Section 4), with the number of overlapping genes highlighted in red. (**B**) Bar plots show *FN1* and *ACTA2* expression levels in HTM cells treated for 24 h with vehicle control, LPA alone, or LPA in combination with 70% EEDK extract and indicated inhibitors. Expression of each gene was normalized to *ACTB*. Statistical significance is indicated (* *p*  <  0.05, ** *p*  <  0.001, and *** *p*  <  0.001; one-way ANOVA). (**C**) Bar plots show AP-1 transactivation after 24 h of treatment with LPA alone or in combination with 70% EEDK extract. Statistical significance is indicated (*** *p*  <  0.001; one-way ANOVA). (**D**) Time-course Western blot analysis of phospho-ERK1/2, ERK1/2, phospho-p38, p38, phospho-JNK, JNK, phospho-c-Jun, c-Jun, phospho-c-Fos, and c-Fos in HTM cells treated with LPA in the absence or presence of 70% EEDK extract. GAPDH was used as a normalization marker. Abbreviation: Ctrl: control.

To elucidate the underlying mechanism of the 70% ethanol EEDK in LPA-induced ECM traits, key transcription factors regulating *ACTA2* and *FN1*, which are closely associated with ECM remodeling, were investigated. Using in silico databases, transcription factors for *ACTA2* (*n* = 8) and *FN1* (*n* = 10) were identified, and seven were found to be common regulators of both genes (Figure 4A). Among these transcription factors, JUN (c-Jun) is a well-known regulator of inflammation and cell proliferation. It is activated downstream of the MAPK pathway and forms heterodimers with c-Fos in the nucleus, constituting the AP-1 complex, which binds to specific promoter elements and functions as a transcription factor [23]. Indeed, inhibitors (e.g., Sorafenib, Wortmannin, U73122, and CC-90003) targeting MAPK-related signaling pathways, significantly suppressed the expression of *FN1* and *ACTA2* in LPA-induced HTM cells, revealing inhibitory effects comparable to those of the 70% ethanol EEDK extract (Figure 4B). Next, an AP-1 binding motif reporter was used to assess transactivation, and the 70% ethanol EEDK extract was found to significantly suppress AP-1 promoter activity compared with LPA-treated cells (Figure 4C). Given that activation and translocation of AP-1, a heterodimer composed of c-Jun and c-Fos, is driven by upstream of MAPK components such as ERK1/2, p38, and JNK, the effect of the 70% ethanol EEDK extract on LPA-induced MAPK activation were next examined. Treatment with the 70% ethanol EEDK extract markedly reduced the phosphorylation of ERK1/2, p38, and JNK in a time-dependent manner compared with cells treated with LPA alone. Consistent with these findings, LPA-induced phosphorylation of c-Jun and c-Fos was also reduced by the 70% ethanol EEDK extract in a time-dependent manner (Figure 4D). Taken together, these findings suggest that the 70% ethanol EEDK extract attenuates LPA-induced ECM remodeling in HTM cells at least in part by inhibiting MAPK-dependent AP-1 activation and the downstream transcription of *ACTA2* and *FN1*.

### 2.6. Protective Effect of 70% EEDK on Retinal Ganglion Cell Survival in a Silicone Oil–Induced Ocular Hypertension Mouse Model

**Figure 5:**
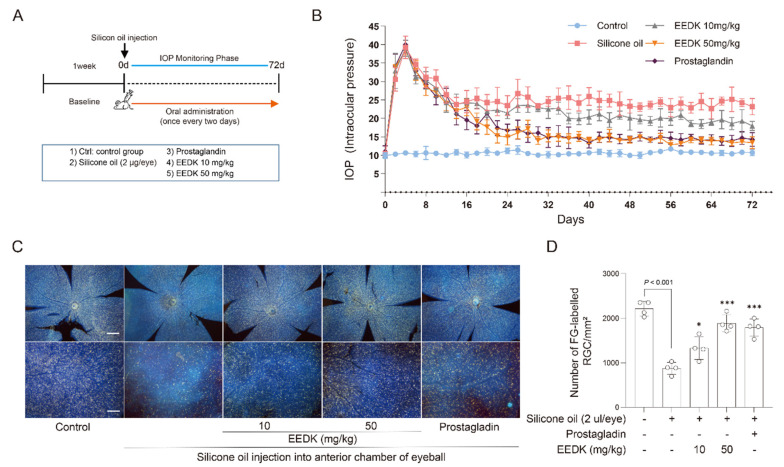
Protective effect of 70% EEDK on retinal ganglion cell survival in a silicone oil–induced ocular hypertension mouse model. (**A**) A schematic overview of the experimental timeline for the silicone oil–induced ocular hypertension mouse model. Prostaglandin (50 μg/mL) was administered as a topical eye drop (one drop daily), whereas 70% EEDK was administered orally once every two days. (**B**) Intraocular pressure changes after intracameral silicone oil injection. Data are presented as mean ± SEM (*n* = 4). (**C**) Representative fluorescence images of retrogradely labeled retinal ganglion cells after intracameral silicone oil injection (scale bars: upper panels, 500 μm; lower panels, 100 μm). (**D**) Quantification of retinal ganglion cell survival expressed as a percentage of the control group. Data are presented as mean ± SEM (*n* = 4). Statistical significance is indicated (* *p*  <  0.05 and *** *p*  <  0.001; one-way ANOVA). Abbreviation: Ctrl: control, IOP: Intraocular pressure.

To investigate the in vivo effects of 70% EEDK, a silicone oil–induced ocular hypertension mouse model was established (Figure 5A). Intracameral injection of silicone oil caused a rapid increase in intraocular pressure (IOP), followed by a gradual decline that nevertheless remained elevated compared with baseline throughout the monitoring period. Oral administration of 70% EEDK (10 or 50 mg/kg) dose-dependently attenuated this sustained IOP elevation, and the 50 mg/kg group exhibited an IOP profile comparable to that of the prostaglandin-treated group (Figure 5B). Consistent with these IOP changes, retrograde labeling showed a marked loss of retinal ganglion cells (RGCs) in the silicone oil-only group, whereas EEDK treatment preserved RGC density, with a more prominent protective effect (Figure 5C). Quantitative analysis confirmed that RGC survival was significantly higher in the EEDK 50 mg/kg and prostaglandin groups than in the silicone oil–only group, while EEDK 10 mg/kg produced a partial improvement (Figure 5D).

## 3. Discussion

Glaucoma is a chromic optic neuropathy characterized by progressive optic nerve damage, for which elevated intraocular pressure (IOP) is the major risk factor [17,24]. Increased IOP is largely attributed to enhanced resistance to aqueous humor outflow (AHO) through the conventional TM pathway [25,26]. Accumulating evidence indicates that fibrotic changes in the TM, such as excessive deposition of ECM components and increased cell migration, contribute to outflow resistance and IOP elevation [7,17,27]. In this study, the mechanisms underlying TM dysfunction were investigated, and the ability of ethanolic extract of EEDK to attenuate TM fibrosis in HTM cells was evaluated.

LPA is a bioactive lipid mediator that has emerged as an important factor in the pathobiology of glaucoma [17,28]. Dysregulated LPA signaling has been implicated in ocular hypertension and other fibrotic disorders [29,30]. LPA is present at substantial levels in serum, extracellular fluid, intracellular compartments, and aqueous humor [14], and activates G protein–coupled LPA receptors that engage Gα subunits and downstream MAPK pathways, including ERK1/2, p38, and JNK [31]. Through these pathways, LPA promotes cell proliferation, survival, and migration [32], supporting its role as a profibrotic mediator in the TM.

Fibrosis is generally characterized by excessive ECM production and accumulation driven by activated mesenchymal-like cells [33,34,35]. TM cells in the juxtacanalicular region exhibit fibroblast-like characteristics and express contractile and ECM proteins such as α-SMA, myosin, various collagens (types I, III, IV, and VI), fibronectin, and laminin [36,37]. In primary open-angle glaucoma, the accumulation of ECM components, including α-SMA and fibronectin, in the TM is thought to hinder aqueous humor outflow and raise IOP [38]. Consistent with these features, the LPA-induced HTM model reproduced key aspects of TM fibrosis, including upregulation of multiple ECM genes (*FN1*, *ACTA2*, *COL1A1*, *COL3A1*, *COL4A1*, *COL6A2*, and *CCN2*) and enhanced cell migration.

EMT is a process in which epithelial cells lose epithelial characteristics and acquire mesenchymal properties, playing a crucial role in tissue development, repair, and remodeling [35,39]. EMT is accompanied by alterations in intercellular adhesion, cell polarity, and migration capacity, and is closely related to fibrotic remodeling [18]. Because the TM is a critical component of the conventional AHO pathway, EMT- and fibrosis-associated ECM deposition in TM cells can increase outflow resistance and ultimately elevate IOP [40]. In our LPA-stimulated HTM model, an EMT-like phenotype was observed, characterized by increased expression of mesenchymal markers, including *CDH2*, *VIM*, and *SNAI1*, together with enhanced cell migration.

A major finding of this study is that pretreatment with 70% ethanol EEDK extract markedly attenuated these LPA-induced profibrotic changes in HTM cells. The 70% ethanol extract reduced the expression of *FN1*, *ACTA2*, *COL1A1*, *COL3A1*, *COL4A1*, *COL6A2*, and *CCN2* in a dose-dependent manner, and significantly inhibited LPA-induced cell migration and wound closure. In contrast, the 90% ethanol EEDK extract did not show comparable antifibrotic effects, suggesting that the active phytochemical constituents are enriched in the 70% ethanol fraction. Together, these results indicate that EEDK exerts a broad antifibrotic effect on the TM by suppressing both ECM production and EMT-like cell motility in response to LPA.

A plausible explanation for the differential efficacy between the 70% and 95% ethanol extracts is that solvent polarity can markedly influence extraction efficiency and the resulting phytochemical composition. In the present HPLC profiling, the 70% ethanol extract exhibited higher peak intensities for several flavonoid glycosides and phenolic acids than the 95% ethanol extract, suggesting an enrichment of more polar glycosylated flavonoids and related phenolics under the 70% ethanol condition. Such compositional differences are consistent with the observation that only the 70% ethanol extract robustly suppressed LPA-induced ECM production and EMT-like migration in HTM cells. Together, these findings support that the anti-fibrotic activity of EEDK is likely driven by a specific phytochemical profile that is more effectively recovered with 70% ethanol than with higher ethanol concentrations.

To explore the underlying mechanism, transcriptional regulation of *ACTA2* and *FN1*, which is closely associated with ECM remodeling, was investigated. In silico analyses identified several transcription factors for *ACTA2* (*n* = 8) and *FN1* (*n* = 10), among which seven factors overlapped as common regulators. Among these, JUN (c-Jun) is a well-known regulator of inflammation and cell proliferation [23]. c-Jun is activated downstream of MAPK signaling and forms heterodimers with c-Fos in the nucleus, constituting the AP-1 complex, which binds to specific promoter elements and regulates ECM- and EMT-related genes [23]. Consistent with this, pharmacological inhibitors targeting MAPK-related signaling pathways, including sorafenib, wortmannin, U73122, and CC-90003, significantly reduced FN1 and ACTA2 expression in LPA-induced HTM cells, showing inhibitory effects comparable to those of the 70% ethanol EEDK extract.

It was further confirmed that 70% ethanol EEDK extract directly interferes with AP-1–dependent transcriptional activity. In AP-1 reporter assays, the extract significantly suppressed LPA-induced AP-1 promoter activity. Given that activation and nuclear translocation of AP-1, a heterodimer composed of c-Jun and c-Fos, are driven by upstream MAPK components such as ERK1/2, p38, and JNK, the effects of 70% ethanol EEDK extract on LPA-induced MAPK activation were examined. Treatment with the extract markedly reduced the phosphorylation of ERK1/2, p38, and JNK in a time-dependent manner compared with LPA alone, and LPA-induced phosphorylation of c-Jun and c-Fos was similarly attenuated. Taken together, these findings suggest that 70% ethanol EEDK extract inhibits LPA-induced TM fibrosis, at least in part, by suppressing MAPK-dependent AP-1 activation and downstream transcription of ECM- and EMT-related genes.

Despite these findings, a limitation of the present study is the use of a crude ethanol extract, rather than isolated individual compounds. However, the primary aim of this work was to evaluate the biological relevance of EEDK as a whole extract, reflecting its traditional use and the potential contribution of synergistic interactions among multiple constituents. Importantly, our results provide a mechanistic basis for the observed antifibrotic effects of EEDK at the cellular signaling level. Future studies will focus on the isolation and structural characterization of individual active constituents responsible for these effects.

The antifibrotic actions of EEDK observed in TM cells are in line with previous reports describing its neuroprotective and IOP-lowering effects in glaucoma models. Pretreatment with an ethanolic extract of persimmon leaves protected retinal ganglion cells (RGCs) from oxidative stress– and excitotoxicity-induced cell death through antioxidant and anti-apoptotic mechanisms [41]. In a mouse model of glaucoma, EEDK also reduced elevated IOP, at least partly by modulating soluble guanylate cyclase α1, a key mediator of vascular hypertension signaling [20]. However, the impact of EEDK on AHO resistance and TM fibrosis has remained unclear. Our findings extend these previous observations by demonstrating that EEDK directly targets LPA/MAPK/AP-1 signaling in HTM cells, thereby suppressing ECM accumulation and EMT-like changes.

Overall, our findings indicate that EEDK may offer multifaceted benefits for glaucoma by promoting RGC survival, influencing IOP-related mechanisms, and reducing fibrotic changes in TM cells. Specifically, the 70% ethanol EEDK extract attenuated LPA-induced profibrotic remodeling in HTM cells by suppressing ECM- and EMT-associated marker expression and inhibiting cell migration. Because the LPA/MAPK/AP-1 axis is conserved across species and implicated in human TM dysfunction, these findings may be relevant to human glaucoma biology; however, generalization to other species and experimental conditions, including in vivo models, remains to be validated. In addition, oral administration of 70% ethanol EEDK reduced sustained IOP elevation and preserved RGC survival in a silicone oil-induced ocular hypertension mouse model. To advance these observations, future studies should (i) confirm antifibrotic efficacy in relevant in vivo models of TM fibrosis, (ii) perform detailed phytochemical profiling to identify active constituents, and (iii) evaluate safety, pharmacokinetics, and therapeutic performance, particularly in combination with standard IOP-lowering treatments.

## 4. Materials and Methods

### 4.1. Preparation of Diospyros kaki Leaves Extraction

*Diospyros kaki* (*D. kaki*) was cultivated in Gangneung, Korea (37.4905° N, 128.5115° E). The leaves were obtained from the Gangneung-si Agricultural Technology Center (Gangneung, Korea), and a voucher specimen (D-521; herbarium no. KN00088J) was deposited at the KIST Gangneung Institute. In this study, *D. kaki* leaves were used as a total (crude) ethanol extract without further fractionation or purification. The collected leaves were air-dried at room temperature under shaded conditions until no further decrease in weight was observed and were subsequently cut into small pieces with a diameter of less than 1 cm. The ethanol extract of *D. kaki* leaves (EEDK) was prepared as previously described [18]. Briefly, 800 g of dried and cut *D. kaki* leaves were extracted three times with 7 L of 70% ethanol (*v*/*v*, Sigma-Aldrich, MilliporeSigma, St. Louis, MO, USA) at room temperature for 3 h in an ultrasonic bath, followed by filtration through Whatman No. 1 filter paper (GE Healthcare, Chicago, IL, USA). The combined filtrates were concentrated to dryness using rotary evaporation at 40 °C and subsequently lyophilized, yielding 33 g of EEDK (4.13%). The EEDK stock solution was prepared in 100% dimethyl sulfoxide (DMSO, Sigma-Aldrich) and stored at −20 °C. For experiments, the stock solution was diluted with cell culture medium to the desired concentrations, with the final DMSO concentration adjusted to 0.5% (*v*/*v*).

### 4.2. HPLC Analysis

HPLC analysis was performed on an Agilent 1200 Infinity HPLC system (Agilent Technologies, Santa Clara, CA, USA) using a YMC Triart C18 column (4.6 × 250 mm, 3 μm; YMC Co., Ltd., Kyoto, Japan). The mobile phase consisted of water containing 0.1% formic acid (Sigma-Aldrich) (A) and acetonitrile containing 0.1% formic acid (Sigma-Aldrich) (B). The flow rate was set at 0.7 mL/min, and the column temperature was maintained at 35 °C. The injection volume was 10 μL, and detection was carried out at 260 nm. The gradient elution program was as follows: 5% B at 0 min, 20% B at 15 min, 20% B at 37 min, 25% B at 45 min, 50% B at 55–60 min, 100% B at 63–67 min, and re-equilibration to 5% B at 70 min. Peak intensities were compared qualitatively between extracts under identical HPLC conditions.

### 4.3. Cell Culture and Drug Treatment

Primary human trabecular meshwork (HTM) cells were purchased from ScienCell Research Laboratories (Carlsbad, CA, USA) and cultured in Trabecular Meshwork Cell Medium (Cat. No. 6591, ScienCell) supplemented with 2% fetal bovine serum (FBS; Cat. No. 0010, ScienCell), 1% Trabecular Meshwork Cell Growth Supplement (Cat. No. 6592, ScienCell), and 1% penicillin–streptomycin solution (P/S; Cat. No. 0503, ScienCell). Cells were incubated at 37 °C in a humidified incubator with 5% CO_2_.

ROCK inhibitors Y-27632 and ripasudil were purchased from Sigma-Aldrich (MilliporeSigma). The RAF inhibitor sorafenib was obtained from Santa Cruz Biotechnology (Dallas, TX, USA). The ERK inhibitor CC-90003 was obtained from Selleck Chemicals (Houston, TX, USA). The PLC inhibitor U-73122 and the PI3K inhibitor wortmannin were purchased from Fisher Scientific (Hampton, NH, USA). Latanoprost, a prostaglandin analog (Xalatan^®^ eye drops), was purchased from Pfizer (New York, NY, USA).

### 4.4. Evaluation of Cell Viability

HTM cells were treated with 12.5–50 μg/mL of EEDK for 24 or 48 h, and cell viability was evaluated using a Quanti-Max™ WST-8 Cell Viability Assay kit (Biomax Co., Ltd., Guri-si, Republic of Korea) according to the manufacturer’s instruction.

### 4.5. Total RNA Extraction and qRT-PCR Analysis

HTM cells were pretreated with EEDK for 2 h and subsequently exposed to LPA for 24 h. Thereafter, total RNA was extracted using the RNeasy Mini Kit (QIAGEN, Germantown, MD, USA), and cDNA was synthesized using the RevertAid First Strand cDNA Synthesis Kit (Thermo Fisher Scientific, Vilnius, Lithuania) according to the manufacturer’s instructions.

For qRT-PCR analysis, 50 ng of cDNA was amplified using a QuantStudio 6 Pro real-time PCR system (Applied Biosystems, Foster City, CA, USA) with TaqMan^®^ Gene Expression Assays (Applied Biosystems) and TaqMan^®^ Fast Advanced Master Mix (Applied Biosystems), as well as AccuPower^®^ 2× GreenStar™ qPCR Master Mix (BIONEER, Daejeon, Republic of Korea) for SYBR Green–based reactions. The information of TaqMan^®^ probes and primer sequences is provided in Table 1.

### 4.6. Western Blotting Analysis

HTM cells were grown in 6-well plates, pretreated with 50 μg/mL EEDK for 2 h, and then treated with 20 μM LPA for either short-term incubations (for analysis of MAPK activation) or long-term incubations (for analysis of ECM proteins and EMT markers). Cells were then lysed in radioimmunoprecipitation assay buffer (Cell Signaling Technology, Danvers, MA, USA) supplemented with a 1× protease inhibitor cocktail and a 1× phosphatase inhibitor cocktail, according to the manufacturer’s instructions.

Whole-cell protein extracts (30 μg per lane) were separated by sodium dodecyl sulfate–polyacrylamide gel electrophoresis using Bolt™ 4–12% Bis–Tris Plus gels (Invitrogen, Carlsbad, CA, USA), transferred onto polyvinylidene difluoride membranes, and probed with epitope-specific primary and secondary antibodies. Bound antibodies were visualized using SuperSignal™ West Femto Maximum Sensitivity Substrate (Thermo Fisher Scientific) and an LAS 4000 imaging system (Fujifilm, Tokyo, Japan).

Monoclonal antibodies against p-ERK1/2 (Thr202/Tyr204), ERK1/2, p-p38 (Thr180/Tyr182), p38, p-JNK (Thr183/Tyr185), JNK, p-c-Jun (Ser73), c-Jun, p-c-Fos (Ser32), c-Fos, E-cadherin, N-cadherin, vimentin, and Snail were purchased from Cell Signaling Technology (Danvers, MA, USA). Polyclonal antibodies against α-SMA, Fibronectin, and GAPDH were purchased from Proteintech (Rosemont, IL, USA).

### 4.7. Wound Healing Assay

Cell migratory capacity was assessed using a wound healing assay. When cells reached about 70–80% confluence, a linear scratch was made in the cell monolayer using a 200 μL pipette tip. After being washed twice with PBS to remove any free-floating cells and debris, the cells were pretreated with or vehicle for 2 h and subsequently exposed to 20 μM LPA for 48 h at 37 °C. Wound closure was observed using an EVOS XL Core microscope (Life Technologies, Grand Island, NY, USA) and quantified with ImageJ 1.53e (National Institutes of Health, Bethesda, MD, USA). Representative images of the wound area for each condition were acquired.

### 4.8. AP-1 Luciferase Reporter Assay

HTM cells seeded in 96-well plates were co-transfected with pAP-1-Luc (BD Biosciences, San Jose, CA, USA) and pNL-Luc (Promega, Madison, WI, USA) plasmids using FuGENE^®^ 6 Transfection Reagent (Roche, Mannheim, Germany) according to the manufacturer’s instructions. Twenty-four hours after co-transfection, the cells were pretreated with 50 μg/mL EEDK for 2 h and then treated with 20 μM LPA for 24 h. The cells were subsequently lysed, and luciferase activity was measured using a Dual-Luciferase^®^ Reporter Assay System (Promega) and a GloMax^®^ Navigator Microplate Luminometer (Promega), following the manufacturer’s instructions. Relative firefly luciferase activity was normalized to NanoLuc^®^ luciferase to account for variations in transfection efficiency. All in vitro experiments were performed in triplicate.

### 4.9. Prediction of Transcription Factors Binding to the FN1 and ACTA2 Promoter Regions Using In Silico Databases

The sequences of *FN1* and *ACTA2* were obtained from the NCBI Gene database (https://www.ncbi.nlm.nih.gov/gene/59 (accessed on 3 January 2024) and https://www.ncbi.nlm.nih.gov/gene/2335 (accessed on 3 January 2024)). To isolate the putative promoter regions of these genes, the sequences were trimmed to encompass positions −2000 to +50 bp relative to the transcription start site (TSS), corresponding to the beginning of exon 1. For comprehensive identification of transcription factors (TFs) associated with these genes, the refined sequences were analyzed using publicly available databases, including GeneCards (https://www.genecards.org/ (accessed on 23 January 2024)) and AliBaba2.1 (http://gene-regulation.com/pub/programs/alibaba2/ (accessed on 23 January 2024)).

ChIP-X Enrichment Analysis version 3 (ChEA3; https://maayanlab.cloud/chea3/ (accessed on 11 February2024)) was used to predict TFs, as previously described [42]. Transcription factor binding sites were further examined using AliBaba2.1 by generating TF binding matrices for *ACTA2* and *FN1* [43]. Specifically, the refined promoter sequences were entered into AliBaba2.1 to generate lists of predicted TFs. These lists were aligned and processed using R (https://www.r-project.org/ (accessed on 11 February2024)) to identify overlapping TFs, which were subsequently ranked in descending order. In addition, the GeneCards database was queried to retrieve TFs associated with *FN1* (2128 TFs from 153 entries) and *ACTA2* (628 TFs from 73 entries). The TFs identified for each gene were compiled, and the overlapping TFs were tabulated and ranked in descending order using R.

### 4.10. Animals and Silicone Oil–Induced Ocular Hypertension Model

Animal experiments were conducted at the KIST Gangneung Institute under specific pathogen-free conditions in accordance with the ARVO Statement for the Use of Animals in Ophthalmic and Vision Research, and all procedures were approved by the Institutional Animal Care and Use Committee of KIST (Approval No. KIST-5088-2022-02-023). Male C57BL/6J mice (6 weeks old, 25–29 g; Central Lab. Animal Inc., Seoul, Korea) were used to establish a silicone oil–induced ocular hypertension model for evaluating the protective effects of EEDK. Mice were acclimated for 1 week prior to experimentation and housed in groups of four with ad libitum access to food and water under controlled conditions (23 ± 0.5 °C, 10% relative humidity, 12 h light/dark cycle; lights on at 07:00). To induce ocular hypertension, mice were anesthetized by intraperitoneal injection of Zoletil and Rompun and placed in a lateral position on a surgical platform; one drop of 0.5% proparacaine hydrochloride (Akorn, Somerset, NJ, USA) was applied topically to the cornea for local anesthesia. A 33-gauge needle was introduced through the superotemporal cornea near the limbus into the anterior chamber while avoiding contact with the iris and lens, and 2 µL of silicone oil (Oxane^®^ 5700; Bausch & Lomb, Rochester, NY, USA) was slowly injected until the oil expanded to cover most of the iris; the micropipette was held in place for 30 s and then carefully withdrawn. To prevent postoperative infection, one drop of 0.5% moxifloxacin ophthalmic solution (Alcon Laboratories Inc., Fort Worth, TX, USA) was applied topically. The contralateral eye served as a control and received an intracameral injection of 2 µL sterile saline. Animals were randomly assigned to groups, and all measurements were performed using the same protocol across groups. No additional controls for treatment/measurement order or cage location were applied. No blinding was performed; group allocation was known to the investigators during treatment administration, outcome measurements, and data analysis. No a priori inclusion or exclusion criteria were established, and no animals/experimental units or data points were excluded from the analyses.

### 4.11. Intraocular Pressure Measurement

Intraocular pressure (IOP) was measured using a TonoLab rebound tonometer (Colonial Medical Supply, Franconia, NH, USA). To minimize diurnal variation, all IOP measurements were performed at the same time of day. For each eye, the device automatically generated an average IOP value from six consecutive measurements after excluding the highest and lowest readings, and the resulting mean was used for subsequent analysis.

### 4.12. RGCs Labeling and Retinal Flat Mount Preparation

The experimental procedures have been described in detail elsewhere [41]. Briefly, mice were subjected to retrograde labeling with Fluoro-Gold (5% in saline; Invitrogen). The tracer was applied to the superior colliculus using a small piece of Gelfoam pre-soaked in the dye. Seven days after application, eyes were enucleated and retinas were dissected. Retinas were detached at the ora serrata, and tissue near the optic nerve head was excised using a trephine. Four radial incisions were made, and retinal flat-mounts were prepared on silane-coated microscope slides.

### 4.13. Statistical Analysis

Results were presented as the mean ± standard deviation from at least three independent experiments. Statistical analyses were performed using GraphPad Prism 10.3.0 (GraphPad Software, San Diego, CA, USA). For comparisons between two groups, a two-tailed unpaired Student’s *t*-test was used. For comparisons among multiple groups, one-way analysis of variance (ANOVA) was used. Normality was assessed using the Shapiro–Wilk test, and homogeneity of variances was assessed using the Brown–Forsythe test. When assumptions were not met, nonparametric tests were applied (Mann–Whitney U test for two-group comparisons and Kruskal–Wallis test for multiple-group comparisons). *p* values < 0.05 were considered statistically significant.

## 5. Conclusions

In conclusion, this study provides that evidence that the 70% ethanol EEDK attenuates LPA-induced fibrotic remodeling in HTM cells by suppressing ECM/EMT marker expression and cell migration, in association with inhibition of MAPK-AP-1 signaling and reduced *ACTA2* and *FN1* transcription. Moreover, oral administration of 70% EEDK alleviated sustained IOP elevation and preserved retinal ganglion cell survival in a silicone oil-induced ocular hypertension mouse model. Collectively, these findings support EEDK as a promising multi-target candidate for glaucoma driven by TM dysfunction.

## Figures and Tables

**Table 1:** Information of SYBR and TaqMan^®^ probes used for qRT-PCR.

Gene Name	Primer Sequence/Assay ID	Assay Type
*ACTA2*	5′-TgT gTg AAg AAg Agg ACA gCA-3′ (sense)	SYBR
	5′-CAC AAT ggA Tgg gAA AAC Ag-3′ (anti-sense)	SYBR
*FN1*	5′-TgT TCg TgC AgC TgT TTA CC-3′ (sense)	SYBR
	5′-CAC TgC ATC CCC ACA gAg TA-3′ (anti-sense)	SYBR
*COL1A1*	5′-TAC AgA ACg gCC TCA ggT ac-3′ (sense)	SYBR
	5′-gCA, gTT CTT ggT CTC gTC AC-3′ (anti-sense)	SYBR
*COL3A1*	5′-AAg AAg gCC CTg AAg CTg AT-3′ (sense)	SYBR
	5′-CCA TTC CCC AgT gTg TTT Cg-3′ (anti-sense)	SYBR
*COL4A1*	5′-ggT ATT CCA ggA TgC AAT gg-3′ (sense)	SYBR
	5′-TCT CAC CTg gAT CAC CCT TC-3′ (anti-sense)	SYBR
*COL6A2*	5′-ggA CgA TgA CCT CAA CTT gC-3′ (sense)	SYBR
	5′-CAg gCg ATg gAg TAg Agg TT-3′ (anti-sense)	SYBR
*CCN2*	5′-gCC TAT TCT gTC ACT TCG gC-3′ (sense)	SYBR
	5′-gCT gCT CTg gAA ggA CTC TC-3′ (anti-sense)	SYBR
*ACTB*	5′-Tgg CAT CCA CgA AAC TAC CT-3′ (sense)	SYBR
	5′-Agg gCA gTg ATC TCC TTC Tg-3′ (anti-sense)	SYBR
*CDH1*	Hs01023894_m1	TaqMan^®^
*CDH2*	Hs00983056_m1	TaqMan^®^
*SNAI1*	Hs00195591_m1	TaqMan^®^
*VIM*	Hs00958111_m1	TaqMan^®^
*GAPDH*	Hs99999905_m1	TaqMan^®^

## Data Availability

The data presented in this study are available on request from the corresponding author. The data are not publicly available due to intellectual property considerations and ongoing patent-related restrictions.

## Supplementary Materials

The following supporting information can be downloaded at: https://www.mdpi.com/article/10.3390/ijms27031544/s1.

## Abbreviations

AHO	aqueous humor outflow
ANOVA	analysis of variance
ChEA3	ChIP-X Enrichment Analysis 3
DMSO	dimethyl sulfoxide
ECM	extracellular matrix
EEDK	ethanolic extract of *Diospyros kaki* leaves
EMT	epithelial–mesenchymal transition
HPLC	high-performance liquid chromatography
HTM	human trabecular meshwork
IOP	intraocular pressure
LPA	lysophosphatidic acid
MAPK	mitogen-activated protein kinase
RGC	retinal ganglion cell
α-SMA	alpha-smooth muscle actin
TGF-β	transforming growth factor-beta
TM	trabecular meshwork
TSS	transcription start site
