# Supplementary material for: Uncovering a Glaucoma-Linked Lysophosphatidic Acid–MAPK/AP-1 Fibrosis Axis in Human Trabecular Meshwork Cells and Its Modulation by Diospyros kaki Leaf Extract
Source: Int J Mol Sci. 2026 Feb 4;27(3):1544. doi: 10.3390/ijms27031544 (PMC12898371; doi:10.3390/ijms27031544)
Supplement: Supplementary file 1 [file ijms-27-01544-s001.zip › Supplementary figures.pdf]

Article

Uncovering a Glaucoma-Linked LPA–MAPK/AP-1 Fibrosis Axis in Human Trabecular Meshwork Cells and Its Modulation by *Diospyros kaki* Leaf Extract

Youngsic Jeon[1,†], Hyukjoon Kwon[1,†], Hong-ryul Ahn[1], Gyuwon Huh[2], Taejung Kim[1,3], Young-Tae Park[1,3], Hyun Bong Park[4], Jin-hyoung Jeong[5], Jae-hyun Jo[6], Young-Joo Kim[1,*], Sang Hoon Jung[1,3,*]

[1] Center for Natural Product Efficacy Optimization, Korea Institute of Science and Technology (KIST), Gangneung Institute of Natural Products, Republic of Korea

[2] R&D Center, NovMetaPharma Co., Ltd., Republic of Korea

[3] Natural Product Applied Science, KIST School, University of Science and Technology, Republic of Korea

[4] Department of Biology, College of Natural Sciences, Gangneung-Wonju National University, Republic of Korea

[5] Department of Healthcare Management, Catholic Kwandong University, Republic of Korea

[6] Department of Digital Healthcare, Catholic Kwandong University, Republic of Korea

[†] These authors contributed equally

Supplementary Figures

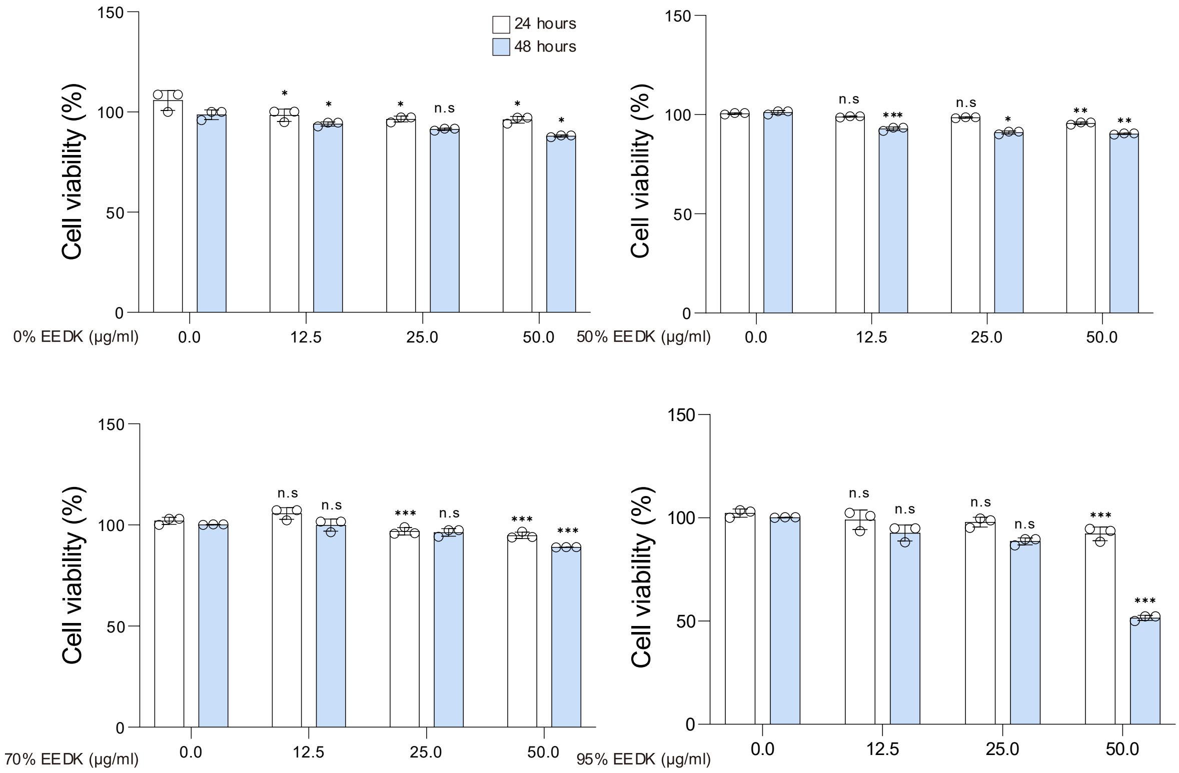

Figure S1. Effects of ethanol-based EEDK extracts on HTM cell viability. Bar plots show HTM cell viability after 24 and 48 h treatment with EEDK extracts prepared using 0%, 50%, 70%, or 95% ethanol. Statistical significance is indicated (*$P < 0.05$, **$P < 0.01$, and ***$P < 0.001$; one-way ANOVA). Abbreviation: *n.s*: not significant.

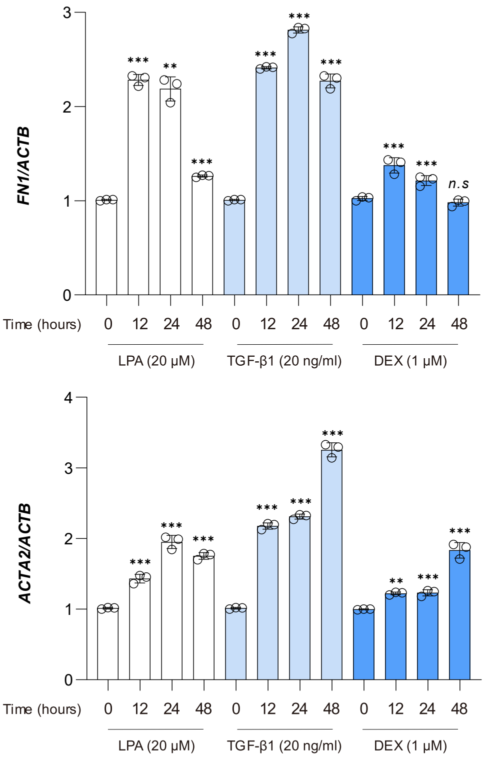

Figure S2. Effects of LPA, TGF-β, and DEX on *FN1* and *ACTA2* expression in HTM cells. Bar plots show FN1 and ACTA2 expression levels in HTM cells treated with LPA, TGF-β, and DEX for 0, 12 h, 24 h, and 48 h. Expression of each gene was normalized to *ACTB*. Statistical significance is indicated (**$P < 0.01$ and ***$P < 0.001$; one-way ANOVA). Abbreviation: LPA: lysophosphatidic acid, TGF-β: transforming growth factor-beta, DEX: dexamethasone, *n.s*: not significant.

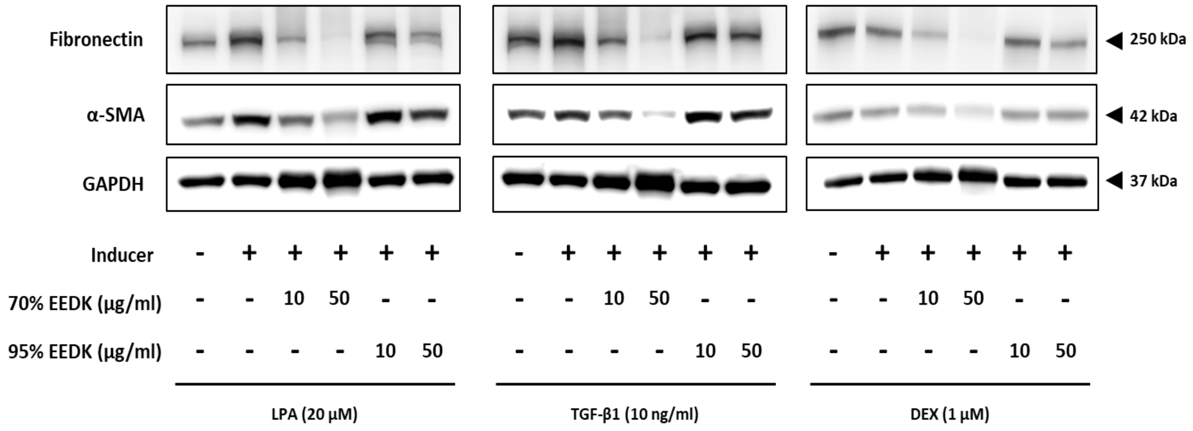

Figure S3. **Effects of LPA, TGF-β, and DEX on fibronectin and α-SMA expression in HTM cells.** Fibronectin and α-SMA protein levels in HTM cells treated with LPA, TGF-β, and DEX for 24 h were assessed by western blot analysis. GAPDH was used as a normalization marker.
